# Effects of interoceptive training on decision making, anxiety, and somatic symptoms

**DOI:** 10.1186/s13030-020-00179-7

**Published:** 2020-03-17

**Authors:** Ayako Sugawara, Yuri Terasawa, Ruri Katsunuma, Atsushi Sekiguchi

**Affiliations:** 1 Department of Behavioral Medicine, National Institute of Mental Health, National Center of Neurology and Psychiatry, 4-1-1 Ogawa-Higashi, Kodaira, Tokyo, 187-8553 Japan; 2 Department of Psychology, Keio University, Minato-ku, Tokyo, Japan

**Keywords:** Interoceptive training, Interoceptive accuracy, Rationality, Decision making, Anxiety, Somatic symptoms

## Abstract

**Background:**

Interoception is the perception of afferent information that arises from any point within the body. Individual differences in interoception have been associated with affective processing and decision-making processing. The somatic marker hypothesis summarizes the potential effects of interoception on decision-making processes. According to this theory, individuals with interoceptive dysfunction exhibit disadvantageous decision making. Recently, enhancement of interoceptive accuracy, an element of interoception assessed by objective decision-making tasks, has been demonstrated using biofeedback. Garfinkle et al. developed an interoceptive training task, modified from the heartbeat perception task, which enhanced interoceptive accuracy and reduced anxiety symptoms. The purpose of this study was to determine the effects of interoceptive training on decision-making processes. Based on improvements in interoceptive accuracy, we hypothesized that decision-making scores would change in a manner indicative of increased rationality.

**Methods:**

This longitudinal interventional study was performed with interoceptive training. Before and after the intervention, interoceptive accuracy and rationality of decision-making processes were assessed using a heartbeat perception task and rational decision-making tasks, respectively. Fourteen healthy volunteers (nine women; mean age, 21.9 ± 4.5 years) participated. The analysis included data from 12 participants. To detect individual differences in the effects of interoceptive accuracy on rationality of decision making, correlation analysis was conducted on change ratios of the indices of interoceptive accuracy and rationality of decision making.

**Results:**

Interoceptive training resulted in significant enhancement of interoceptive accuracy scores and significant reductions in somatic symptom and state anxiety scores. In contrast, interoceptive training did not cause significant changes in decision-making indices. There was a significant positive correlation between change ratios of indices of interoceptive accuracy and rationality of decision making.

**Conclusions:**

The results suggested a causal relation between interoception and rationality of decision making. These findings will enhance the understanding of mechanisms underlying alterations of decision-making related to psychotherapy by focusing on interoception.

**Trial registration:**

Trial registration number: UMIN000037548.

## Background

Interoception is the perception of afferent information that arises from any point within the body [1, 2]. Interoceptive signaling from the body to the brain involves several pathways, including the hormonal, immune, and autonomic nervous systems [2]. Interoception is associated with emotional experiences and somatic symptoms, and reflects sensitivity to somatic symptoms. Since the 1960s, it has been widely accepted that somatic perceptions affect emotional feelings [3]. For example, normal participants with enhanced interoceptive accuracy had intense emotional experiences [4], and scores on an index of interoceptive accuracy and somatic perception were correlated with anxiety scores in healthy participants [5, 6]. Moreover, patients with anxiety disorder tended to have heightened interoceptive accuracy [7].

Interoceptive dysfunction has been observed in several types of stress-related disorders with psychological and somatic symptoms, including panic disorders, somatic symptom disorders, and substance use disorders [8]. Data from past studies indicated that patients with major depressive disorder are hypersensitive to body signals [9, 10]; a similar finding was reported in patients with irritable bowel syndrome [11]. In contrast, another study indicated that patients with depressive disorder had reduced interoceptive accuracy [12]. In addition, patients with anorexia and patients with bulimia nervosa showed hypo- and hypersensitivity to cues of hunger, respectively [13, 14]. Furthermore, patients with disorders characterized by somatic symptoms exhibited impaired interoceptive accuracy [15]. Thus, both hypo- and hypersensitive interoception are associated with stress-related diseases.

Recently, interoceptive accuracy was shown to be enhanced by a cognitive training task using a biofeedback technique modified from a heartbeat discrimination task [16–18]. In the heartbeat discrimination task, participants were presented with a series of tones that were presented either in a manner corresponding to their own heartbeat (synchronous condition) or with a delay (asynchronous condition). Garfinkel et al. developed a cognitive training task to enhance interoceptive accuracy by modifying the heartbeat discrimination task with additional immediate correct or incorrect feedback for participants in each trial, with the aim of training heartbeat perception. The interoceptive accuracy was assessed using a heartbeat perception task [19] as an index of cardiac perception. Although interoceptive training involving cardiac perception enhanced cardiac perception itself, there was no evidence of a transfer effect of interoceptive training involving cardiac perception to other organs or sympathetic activity during a gambling task. In their pilot study, Garfinkel et al. demonstrated that eight sessions of interoceptive training enhanced interoceptive accuracy and reduced anxiety symptoms in healthy individuals [20].

Individual differences in interoceptive accuracy have been associated with anxiety symptoms, as well as decision-making processes [21]. The somatic marker hypothesis suggested that interoception affects decision-making processes and that these processes are impaired by lesions in the ventromedial prefrontal cortex [22]. Specifically, Damasio et al. presented two types of options in a gambling task: low-risk, low-return decks and high-risk, high-return decks. Participants did not know which decks were advantageous at the start of the task, but usually noticed which option carried higher risk. However, before choosing the option with higher risk, participants exhibited increased skin conductance, reflecting sympathetic nervous activity. This phenomenon was present before participants realized which option carried higher risk. Given that sympathetic nervous activity is a main interoception pathway [2], these findings suggested that rational decision making was preceded by interoception. Notably, participants with interoceptive dysfunction were shown to select the disadvantageous option in a similar study paradigm [23]. Furthermore, participants with increased interoceptive accuracy were likely to exhibit adaptive intuitive decision making [24]. Therefore, we concluded that interoceptive training could affect the rationality of decision making.

The original purpose of this study was to determine the effects of interoceptive training on decision making. The study also examined the effects of interoceptive training on somatic symptoms and anxiety levels, which had been reported in a previous study [20]. We hypothesized that interoceptive training would reduce anxiety levels and somatic symptoms and that, based on improvements in interoceptive accuracy, decision-making scores would change in a manner indicative of increased rationality.

## Methods

### Participants

Fourteen healthy volunteers (nine women; mean age, 21.9 ± 4.5 years) participated in this study; the volunteers were recruited from among graduate and undergraduate university students. No participants had a history of psychiatric disorders. Data were omitted for one participant who did not perform the intervention task for personal reasons. Data from another participant were unavailable because of an unexpected technical error in the tablet PC. Thus, the analysis included data from 12 participants. Each participant provided written informed consent for inclusion in the study. The study protocol was approved by the ethics committee of the National Center of Neurology and Psychiatry (A2018–013) and was registered in the University Hospital Medical Information Network (UMIN) Clinical Trials Registry (URL: http://www.umin.ac.jp), No. UMIN000037548.

### Procedure

The interoceptive training programs were developed in-house using matlab2012a and were installed on a personal computer for use by the participants. Participants were asked to complete at least four training sessions in 1 week, in accordance with the protocol used in a prior study [20]. Each training session was approximately 40 min, but the total time was dependent on self-pacing intervals between trials. All participants underwent psychological and behavioral assessments before and after the 1-week training period.

### Interoceptive training task

**Fig. 1:**
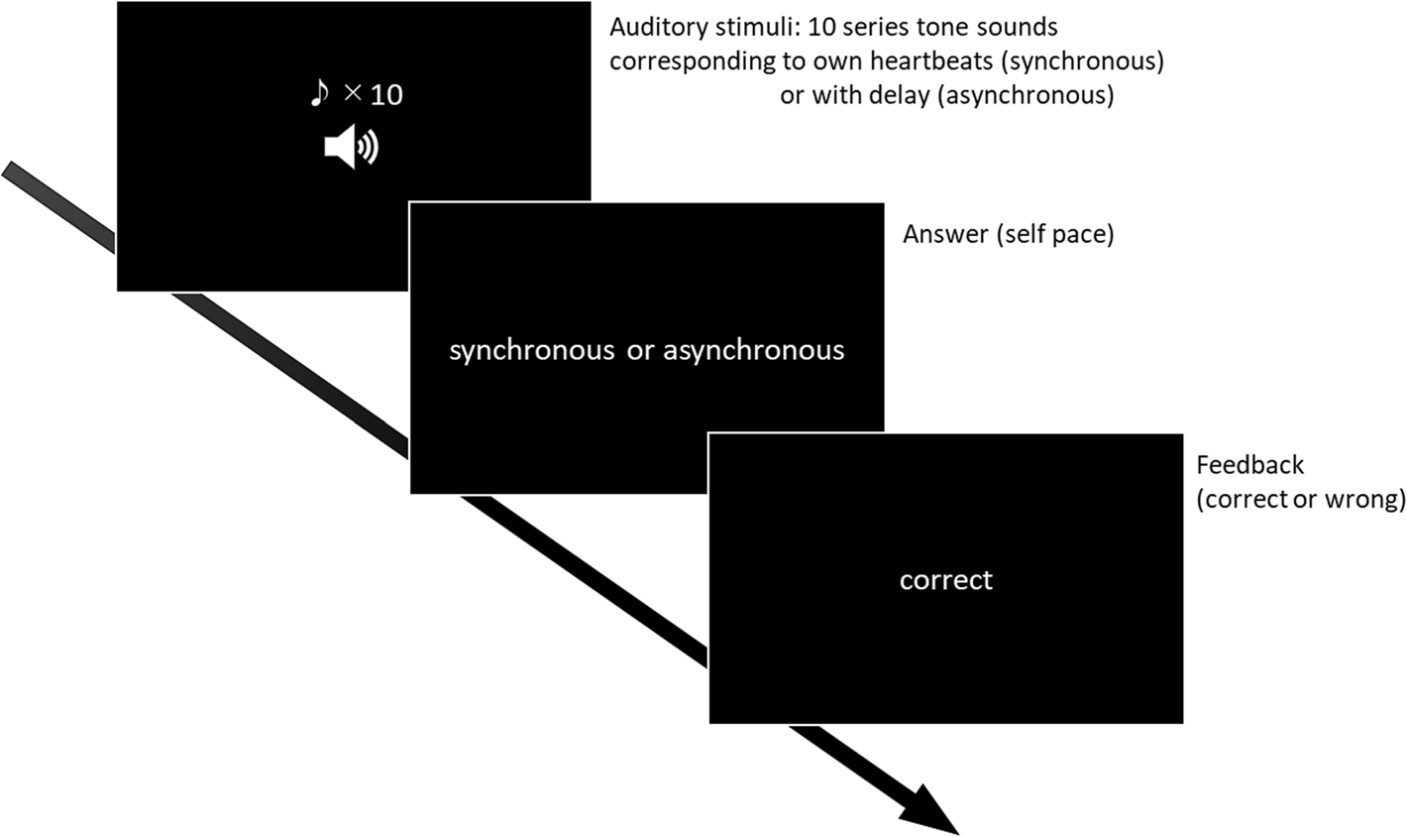
Interoceptive training task

The interoceptive training task consisted of a modified heartbeat discrimination task [16–18]. In this task, participants were presented with a series of tones that were presented either in a manner corresponding to their own heartbeat (synchronous condition) or with a delay (asynchronous condition) (Fig. 1). Each trial consisted of 10 100-ms tones presented at 440 Hz, triggered by the participant’s own heartbeat, which was monitored by a pulse meter attached to the index finger. Under the synchronous condition, tones were generated at the beginning of the rising edge of the pressure wave; under the asynchronous condition, a delay of 300 ms was included in the presentation. In accordance with the method used by Garfinkel et al. [20], to update subjects’ heartbeat perception, we added immediate feedback at the end of each trial to indicate whether their responses were correct or incorrect. The task consisted of 80 trials in a single daily session. The instructions for participants were as follows: “You will hear 10 sequential tone sounds that are associated with your own heartbeat in each trial. The tone sounds in some trials are synchronous with your heartbeat. Those in other trials are asynchronous with your heartbeat because of a short time delay. You must focus on your own heartbeat without taking your pulse during the entire trial. After each trial, you will be asked to determine whether tone sounds were synchronous or asynchronous with your own heartbeat.”

### Psychological assessments

#### Anxiety symptoms

Anxiety symptoms were evaluated using the State–Trait Anxiety Inventory [25, 26]. This self-reported questionnaire consisted of 40 items to measure state anxiety and trait anxiety using normal and reversed four-point Likert scales, where higher scores indicated greater anxiety.

#### Somatic symptoms

Somatic symptoms in daily life were assessed using the modified somatic perception questionnaire [27]. This questionnaire consisted of 22 items designed to evaluate how participants felt during the past 1 week with respect to somatic symptoms, including increased heart rate, the sensation of a pulse in the neck, the sensation of butterflies in the stomach, pain or ache in the stomach, difficulty in swallowing, and dryness in the mouth. Participants were asked to provide responses using four-point Likert scales, where higher scores indicated more sensitive somatic perception.

### Behavioral assessments

#### Interoceptive accuracy

Interoceptive accuracy was estimated using a heartbeat perception task [19]. Participants were asked to count their heartbeat three times during certain periods (25 s, 35 s, and 45 s) without taking their pulse, while their actual heartbeat was recorded by a pulse meter. The order of trials was not shuffled among subjects because of a technical limitation of the program running on a tablet PC. By using both measures of heartbeat, interoceptive accuracy (IA) scores were calculated with the following formula:

$$ \mathrm{interoceptive}\ \mathrm{accuracy}\ \mathrm{score}=1/3\sum \left\{1-\left(|\mathrm{recorded}\ \mathrm{count}-\mathrm{counted}\ \mathrm{count}|/\mathrm{recorded}\ \mathrm{count}\right)\right\} $$

#### Rationality of decision making

We did not use the decision-making task described by Damasio [22] to estimate the rationality of participants’ decision making because this task was inappropriate for our longitudinal study because of the expected learning effect. Damasio’s gambling task required subjects to choose advantageous decks to earn greater amounts of money; nearly all healthy subjects were able to discern which decks were advantageous during the latter half of the task. We presumed that most participants could easily detect which decks were advantageous in the second assessment, despite the use of a 1-week interval.

**Fig. 2:**
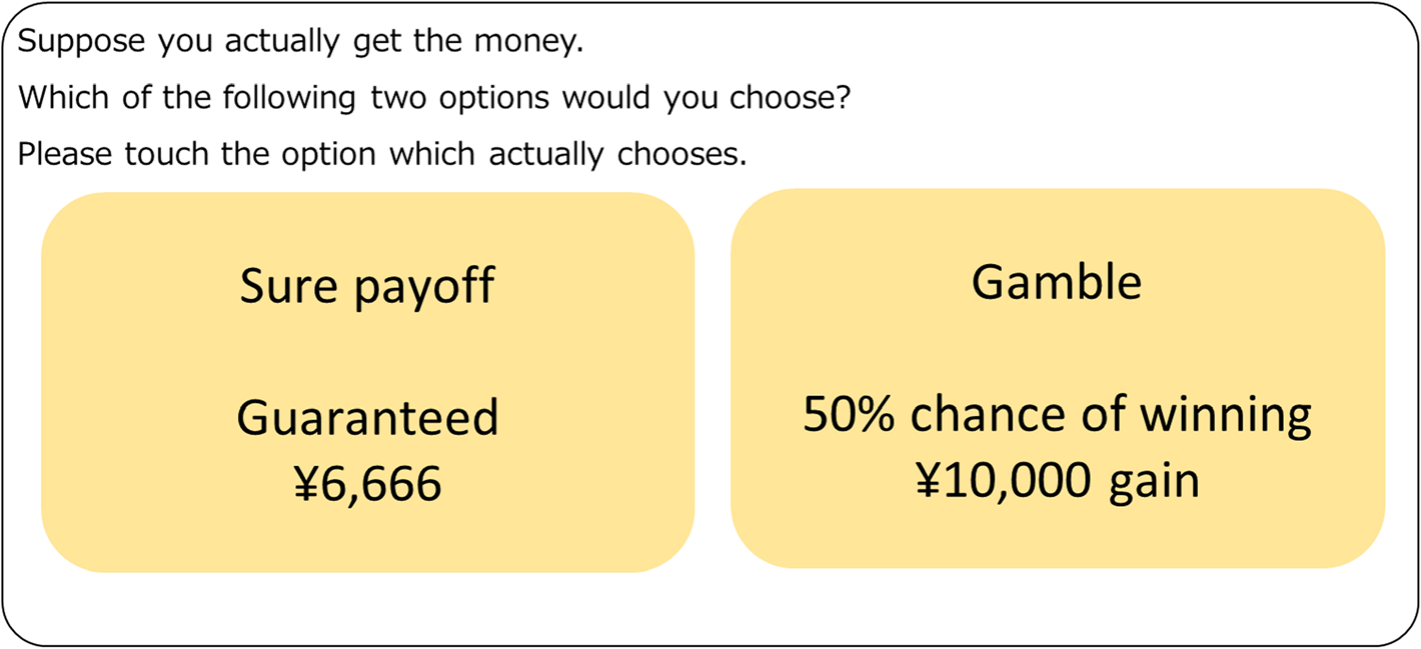
Decision-making task. An example of a choice between gambling and sure payoff on a tablet monitor. Gambling was presented with a certain probability *p* (*p* = 5, 10, 30, 50, 80, and 95%) and a fixed result of ¥10,000. Participants had to press the right or left button to choose gambling or sure payoff, in accordance with their preference. The positions (left and right) of the two options were randomized

In the present study, we used the following monetary reward task, modified from a previous experiment [28]. Participants were shown two options on the screen of a tablet PC: sure payoff and a gamble. Gambles were presented with the objective probability *p* of paying a known monetary reward *X* and paying zero otherwise, such as “*p*% chance of winning *X* (gamble), and gaining *Y* (sure payoff).” X was fixed as 10,000 yen in all trials. The following *p*-values were set: 5, 10, 30, 50, 80, and 95. There were a maximum of eight gambles for each *p*. The order of gambles was randomized across participants. Instructions for participants were as follows: “Two options for possible monetary gain will be presented to you. Option 1 is a sure payoff, and option 2 is a gamble. For example, you will see a guaranteed payoff of 6,666 yen on one side of the monitor; on the other side, you will see a gamble in which you have a 50% chance of winning 10,000 yen. Make a choice between the two options based on your preference by pressing the right or left button. There is no correct answer and no time limit. After you make a choice, subsequent options will be presented.” This approach was similar to the method used by Takahashi et al. [28] (Fig. 2). The amount of monetary reward for a sure payoff was adjusted based on choices in prior trials. After eight trials per gamble, a certainty equivalent was calculated for each *p*. The rules for adjustment and the calculation of the certainty equivalent were in accordance with the method used in a previous study [28].

**Fig. 3:**
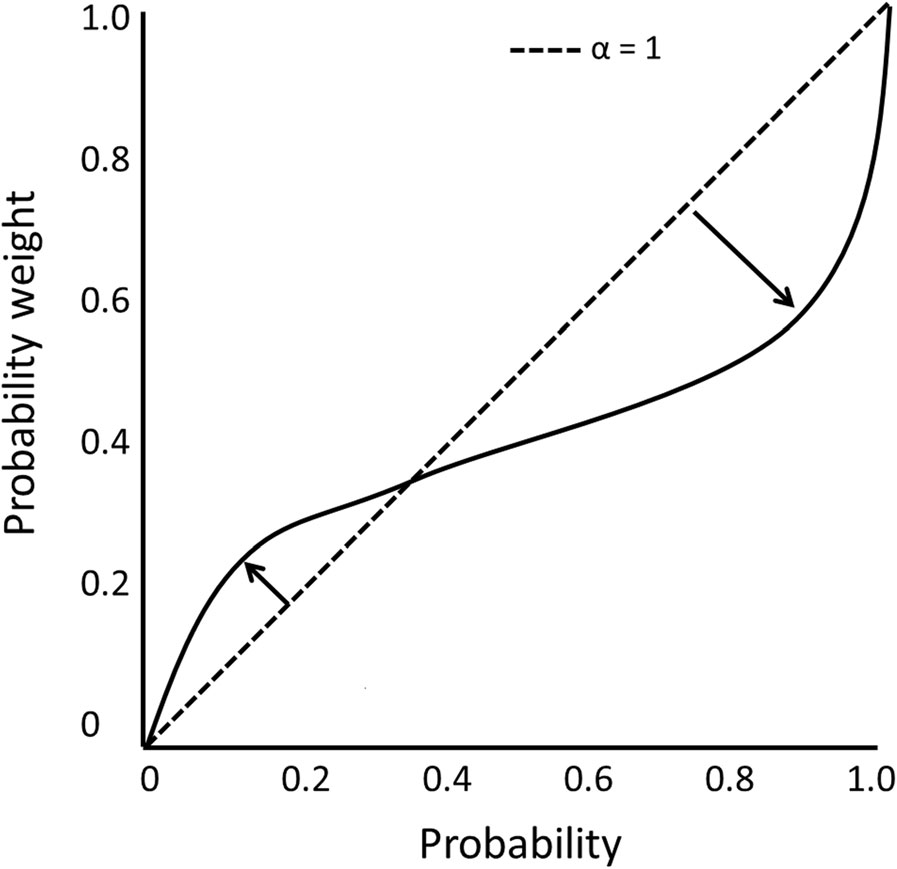
Nonlinear weighting of probability inferred from choices. Fitted probability weighting function using the Prelec model

Next, we estimated the index of rationality of decision making for each participant. The method of estimation was in accordance with the method used in a previous study [28]. In accordance with prospect theory [29], we used a single parameter function derived by Prelec [30], w(p) = exp.{−(ln(1/p))α} with 0 < α < 1, where w is the decision weight of each *p*. In this function, the degree of nonlinearity is explained by a single parameter (α) for each participant. This w(p) function has an inverted “S” shape (Fig. 3). The parameter α indicates the degree of nonlinearity in a mathematical manner. In this experiment, the α score was estimated to indicate rationality for each participant. A smaller value of α represented a more nonlinear inflected weighting function and a higher value represented a more linear weighting function. The weighting function and utility function were estimated by the least-squares method.

**Fig. 4:**
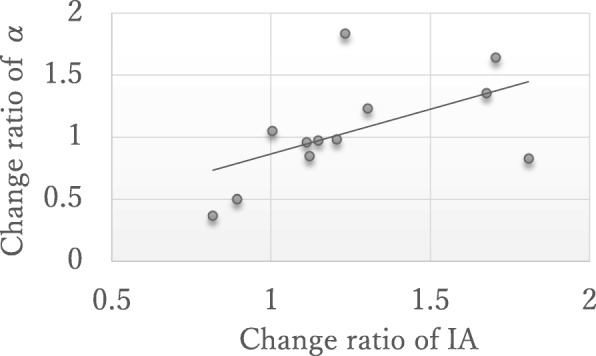
Positive correlation between change ratio of alpha and IA. Pearson’s r = 0.55, *p* < 0.05. IA; interoceptive accuracy

### Data analyses

Psychological and behavioral data were analyzed using SPSS v.25 software (IBM Corp., Armonk, NY, USA). To detect significant longitudinal changes caused by training, paired *t* tests were conducted regarding indices of interoceptive accuracy and rationality of decision making, as well as regarding scores for anxiety and somatic symptoms. With regard to indices of interoceptive accuracy, rationality of decision making, and state anxiety levels, statistical thresholds were set at one-tailed *p* = 0.05, in accordance with our hypothesis and previous findings that indices of interoceptive accuracy increased after training, whereas state anxiety levels decreased [20]. For scores of trait anxiety and somatic symptoms, statistical thresholds were set at two-tailed *p* = 0.05. To detect individual differences in the effects of interoceptive accuracy on rationality of decision making, correlation analysis was conducted on the change ratios for interoceptive accuracy scores and α scores. Change ratio was defined as the score after training divided by the baseline score. Statistical thresholds were set at one-tailed *p* = 0.05, in accordance with our hypothesis that decision-making scores would become more rational with improved interoceptive accuracy.

## Results

Training resulted in a significant increase in interoceptive accuracy score (*p* = 0.007) and a significant reduction in state anxiety score (*p* = 0.04), as well as a marginally significant reduction in somatic perception symptoms (*p* = 0.054, Table 1). In contrast, training did not result in significant changes in trait anxiety score (*p* = 0.29) or the index of rationality of decision making (*p* = 0.31, Table 1). However, there was a significant positive correlation between the change ratio of interoceptive accuracy scores and the change ratio of α scores (Pearson’s r = 0.55, *p* = 0.03, Fig. 4). This positive correlation indicated that participants with enhanced interoceptive accuracy were more likely to show a change toward more rational decision making.

**Table 1:** Psychological changes

Psychological measures (***n*** = 12)	Before training	After training	
IA	0.70 ± 0.15	0.84 ± 0.11	*p* = 0.007 ^a^
α (rationality)	0.75 ± 0.15	0.81 ± 0.33	*p* = 0.31 ^a^
Somatic symptoms	5.0 ± 3.8	3.5 ± 2.9	*p* = 0.054 ^b^
State anxiety (STAI)	17.5 ± 7.0	15.3 ± 7.7	*p* = 0.04 ^a^
Trait anxiety (STAI)	24.5 ± 10.0	23.8 ± 9.1	*p* = 0.29 ^b^

STAI, State–Trait Anxiety Inventory

^a^ one-tailed paired *t* test

^b^ two-tailed paired *t* test

## Discussion

After 1 week of interoceptive training, the participants in this study exhibited enhanced interoceptive accuracy and reductions in both somatic symptoms and anxiety level; furthermore, their decision-making processes shifted in a more rational direction compared with baseline. The results suggested a causal relation between interoception and rationality of decision making. These findings support our hypotheses that interoceptive training would reduce somatic and anxiety symptoms and that, based on improvements in interoceptive accuracy, decision-making scores would change in a manner indicative of increased rationality.

Enhanced interoceptive accuracy after training seems to represent a direct training effect. Because of the single-arm experimental design, some other interpretations are possible, such as a learning effect in the heartbeat detection task or an effect of increased body awareness among participants. However, our participants did not receive feedback regarding their heartbeat count during the heartbeat perception task, and therefore we considered a learning effect to be relatively less likely. Furthermore, a previous study did not show a learning effect in the heartbeat perception task with 1-week interval training, even in a no-intervention control group [31]. The results of that study also showed that 15 min of body scanning over a 1-week period did not improve heartbeat detection [31], suggesting that increased body awareness did not improve interoceptive accuracy. Therefore, we concluded that our 1-week interoceptive training led to improved interoceptive accuracy in healthy participants.

There is some controversy regarding the relation between interoception and clinical symptoms, such as anxiety levels and somatic symptoms; however, the concept of multiple elements of interoception [32], which was consistent with our observations, may provide a solution to this controversy. The relation between interoceptive accuracy and anxiety symptoms in patients with anxiety apparently differs from the relation in healthy individuals. Patients with anxiety were reported to show increased interoceptive accuracy [7]. Based on the findings in patients with anxiety, elevated interoceptive accuracy was expected to increase somatic perception and anxiety levels. Conversely, enhancement of interoceptive accuracy by interoceptive training resulted in reduced state anxiety scores in healthy individuals [20]. Consistent with the results of Garfinkel’s study, anxiety levels were reduced by interoceptive training in our healthy participants. Based on the concept of multiple elements of interoception (i.e., interoceptive awareness, interoceptive accuracy, and interoceptive sensibility) [32], interoceptive accuracy is assessed by an objective behavioral test, such as the heartbeat perception task, and interoceptive sensibility is measured by somatic symptoms in daily life using a self-reported questionnaire, such as the modified somatic perception questionnaire [27] (used in our study) or the Multidimensional Assessment of Interoceptive Awareness [33, 34]. Garfinkel et al. proposed that discrepancies between interoceptive accuracy and sensibility would cause anxiety and somatic symptoms [32, 35]. According to this concept, for participants with lower interoceptive accuracy, rather than interoceptive sensibility, interoceptive accuracy training would reduce their anxiety and somatic symptoms due the resultant decrease in dissociation. Applying this concept, the enhancement of interoceptive accuracy and reductions of anxiety levels and somatic symptoms in healthy participants in the present study represented a smaller discrepancy between interoceptive accuracy and sensibility. The results presented here support the hypothesis described in healthy individuals, while further studies are required in subjects with anxiety.

A causal relation (i.e., interoceptive accuracy influences decision-making processing) would suggest that psychotherapy affects behavioral alteration, as well as the dopamine system. As expected, individuals with enhanced interoceptive accuracy after interoceptive training were more likely to show changes in their decision-making scores that were indicative of increased rationality. As there was no significant change in the mean index of decision-making rationality due to training, the positive correlation between change ratios of indices of interoception and rationality of decision making was affected by individual differences in the effects of training. An association between enhancement of interoception and alteration of rational decision making was established in past studies. Participants with increased interoceptive accuracy were likely to have adaptive intuitive decision making [24], as well as inhibitory behaviors [36]. Furthermore, the index of rationality of decision making was related to dopamine receptor density in the striatum [28]. The results of the present intervention study indicated a causal relation between interoceptive accuracy and decision-making processing. These findings provide a better understanding of the mechanism underlying changes in decision making due to psychotherapy focused on interoception. Indeed, interoceptive exposure therapy for panic disorder [37] and irritable bowel syndrome [38, 39] led to reductions in anxiety and somatic symptoms, respectively. In addition, mindfulness-based stress reduction is often regarded as a component of contemplative interoception training [40]; this type of training was presumed to influence changes in decision making. Alterations of the dopaminergic system in the brain may also contribute to the abilities of these psychotherapies to induce adaptive decision making.

This study has some limitations. First, as described earlier, our interpretation of training effects was not conclusive because of the study’s single-arm design. To detect a learning effect or more conclusively establish a training effect, appropriate control groups are needed. Second, the sample size in this study was relatively small, which may have impacted our ability to detect a significant change in the index of rationality of decision making. Third, because our participants were healthy volunteers, further studies in patients with stress-related disorders are needed.

## Conclusion

The results of the present study suggest a causal relation between interoception and the rationality of decision making. These findings provide the first evidence of changes in decision making due to psychotherapy focused on interoception. Notably, the neurological background underlying interoception and rationality of decision making has been well examined [5, 6, 28, 41–44]; therefore, future studies should include investigations of the neurological underpinnings of this causal relation.

## Data Availability

Data from participants who agreed to the public distribution of data are available from the corresponding author upon reasonable request.

## Abbreviations

IA: interoceptive accuracy
STAI: State–Trait Anxiety Inventory
